# Supplementary material for: Multi‐dataset identification of innovative feature genes and molecular mechanisms in keratoconus
Source: J Cell Mol Med. 2024 Sep 19;28(18):e70079. doi: 10.1111/jcmm.70079 (PMC11412914; doi:10.1111/jcmm.70079)
Supplement: Supplementary file 2 — Material S2. [file JCMM-28-e70079-s002.zip › 7.GSEA analysis/3.DBNDD1/KEGG_CHEMOKINE_SIGNALING_PATHWAY.html]

Details for gene set KEGG\_CHEMOKINE\_SIGNALING\_PATHWAY[GSEA]

|  || Dataset | DBNDD1.group.cls#h\_versus\_l |
| Phenotype | group.cls#h\_versus\_l |
| Upregulated in class | l |
| GeneSet | KEGG\_CHEMOKINE\_SIGNALING\_PATHWAY |
| Enrichment Score (ES) | -0.3971288 |
| Normalized Enrichment Score (NES) | -1.4081892 |
| Nominal p-value | 0.0501002 |
| FDR q-value | 0.6373731 |
| FWER p-Value | 0.889 |
Table: GSEA Results Summary

  

Fig 1: Enrichment plot: KEGG\_CHEMOKINE\_SIGNALING\_PATHWAY      
 Profile of the Running ES Score & Positions of GeneSet Members on the Rank Ordered List

  

| SYMBOL | TITLE | RANK IN GENE LIST | RANK METRIC SCORE | RUNNING ES | CORE ENRICHMENT || 1 | PIK3R5 | na | 35 | 0.708 | 0.0337 | No |
| 2 | CXCR2 | na | 3338 | 0.238 | -0.0502 | No |
| 3 | CCR10 | na | 4137 | 0.209 | -0.0631 | No |
| 4 | HCK | na | 4340 | 0.202 | -0.0590 | No |
| 5 | CCL2 | na | 4419 | 0.199 | -0.0515 | No |
| 6 | PF4 | na | 4760 | 0.188 | -0.0521 | No |
| 7 | JAK3 | na | 5505 | 0.164 | -0.0656 | No |
| 8 | GNG3 | na | 5883 | 0.154 | -0.0690 | No |
| 9 | ARRB1 | na | 5893 | 0.153 | -0.0617 | No |
| 10 | ADCY7 | na | 6033 | 0.150 | -0.0584 | No |
| 11 | CCR2 | na | 6130 | 0.147 | -0.0540 | No |
| 12 | PPBP | na | 6173 | 0.146 | -0.0481 | No |
| 13 | ADCY9 | na | 6468 | 0.137 | -0.0498 | No |
| 14 | ADCY6 | na | 6632 | 0.133 | -0.0480 | No |
| 15 | SHC3 | na | 6808 | 0.129 | -0.0468 | No |
| 16 | RASGRP2 | na | 6889 | 0.127 | -0.0429 | No |
| 17 | PIK3R1 | na | 6912 | 0.126 | -0.0373 | No |
| 18 | BCAR1 | na | 7587 | 0.112 | -0.0513 | No |
| 19 | GNG8 | na | 7762 | 0.108 | -0.0511 | No |
| 20 | CCL26 | na | 7877 | 0.106 | -0.0492 | No |
| 21 | ELMO1 | na | 7980 | 0.104 | -0.0470 | No |
| 22 | ADCY4 | na | 8335 | 0.097 | -0.0525 | No |
| 23 | ADCY5 | na | 8470 | 0.095 | -0.0517 | No |
| 24 | SOS1 | na | 8586 | 0.093 | -0.0505 | No |
| 25 | PLCB2 | na | 9009 | 0.086 | -0.0585 | No |
| 26 | AKT2 | na | 9388 | 0.080 | -0.0655 | No |
| 27 | TIAM2 | na | 9526 | 0.078 | -0.0657 | No |
| 28 | CCL15 | na | 9778 | 0.074 | -0.0693 | No |
| 29 | MAPK3 | na | 9865 | 0.073 | -0.0682 | No |
| 30 | CCL23 | na | 10022 | 0.071 | -0.0692 | No |
| 31 | CCL3L3 | na | 10246 | 0.068 | -0.0723 | No |
| 32 | CCR4 | na | 10530 | 0.064 | -0.0774 | No |
| 33 | VAV2 | na | 10635 | 0.063 | -0.0773 | No |
| 34 | CSK | na | 11685 | 0.050 | -0.1052 | No |
| 35 | PIK3R2 | na | 11699 | 0.050 | -0.1031 | No |
| 36 | CXCR5 | na | 11759 | 0.050 | -0.1024 | No |
| 37 | ROCK1 | na | 11947 | 0.048 | -0.1055 | No |
| 38 | XCR1 | na | 12568 | 0.042 | -0.1214 | No |
| 39 | CCL17 | na | 13259 | 0.036 | -0.1396 | No |
| 40 | SOS2 | na | 13580 | 0.033 | -0.1472 | No |
| 41 | GRK6 | na | 13765 | 0.032 | -0.1510 | No |
| 42 | CCR6 | na | 13914 | 0.031 | -0.1538 | No |
| 43 | CCL11 | na | 14105 | 0.029 | -0.1578 | No |
| 44 | KRAS | na | 14127 | 0.029 | -0.1570 | No |
| 45 | PRKCZ | na | 14131 | 0.029 | -0.1557 | No |
| 46 | PIK3R3 | na | 14144 | 0.029 | -0.1546 | No |
| 47 | GNAI3 | na | 14246 | 0.028 | -0.1561 | No |
| 48 | GNG10 | na | 14454 | 0.027 | -0.1608 | No |
| 49 | CCR5 | na | 14673 | 0.025 | -0.1659 | No |
| 50 | RAC1 | na | 14759 | 0.025 | -0.1672 | No |
| 51 | GNG7 | na | 14929 | 0.023 | -0.1709 | No |
| 52 | GNAI2 | na | 15169 | 0.022 | -0.1768 | No |
| 53 | CXCL5 | na | 15230 | 0.021 | -0.1775 | No |
| 54 | BRAF | na | 15471 | 0.019 | -0.1835 | No |
| 55 | GNB2 | na | 15485 | 0.019 | -0.1829 | No |
| 56 | PRKX | na | 15799 | 0.017 | -0.1911 | No |
| 57 | STAT5B | na | 16077 | 0.015 | -0.1984 | No |
| 58 | PARD3 | na | 16208 | 0.014 | -0.2014 | No |
| 59 | VAV3 | na | 16809 | 0.011 | -0.2183 | No |
| 60 | RAP1A | na | 16884 | 0.010 | -0.2199 | No |
| 61 | CCL4L2 | na | 16968 | 0.010 | -0.2219 | No |
| 62 | PIK3CA | na | 17073 | 0.009 | -0.2244 | No |
| 63 | GNB1 | na | 17142 | 0.008 | -0.2260 | No |
| 64 | GNB5 | na | 17534 | 0.006 | -0.2370 | No |
| 65 | CCL18 | na | 17870 | 0.004 | -0.2465 | No |
| 66 | RAP1B | na | 18018 | 0.003 | -0.2507 | No |
| 67 | WAS | na | 18085 | 0.002 | -0.2525 | No |
| 68 | PIK3CB | na | 18338 | 0.000 | -0.2597 | No |
| 69 | ARRB2 | na | 18679 | -0.002 | -0.2695 | No |
| 70 | PF4V1 | na | 18854 | -0.003 | -0.2744 | No |
| 71 | CHUK | na | 18953 | -0.004 | -0.2770 | No |
| 72 | HRAS | na | 18984 | -0.004 | -0.2776 | No |
| 73 | RAF1 | na | 19005 | -0.005 | -0.2780 | No |
| 74 | GSK3A | na | 19059 | -0.005 | -0.2793 | No |
| 75 | AKT1 | na | 19066 | -0.005 | -0.2792 | No |
| 76 | RHOA | na | 19096 | -0.005 | -0.2798 | No |
| 77 | FGR | na | 19118 | -0.005 | -0.2802 | No |
| 78 | ADCY2 | na | 19167 | -0.006 | -0.2813 | No |
| 79 | PLCB3 | na | 19605 | -0.010 | -0.2934 | No |
| 80 | TIAM1 | na | 19651 | -0.010 | -0.2943 | No |
| 81 | PLCB4 | na | 19707 | -0.010 | -0.2953 | No |
| 82 | STAT1 | na | 19966 | -0.013 | -0.3022 | No |
| 83 | WASL | na | 20105 | -0.014 | -0.3055 | No |
| 84 | GNG5 | na | 20203 | -0.015 | -0.3076 | No |
| 85 | GNG13 | na | 20606 | -0.018 | -0.3183 | No |
| 86 | CCL24 | na | 20660 | -0.019 | -0.3189 | No |
| 87 | PRKCD | na | 20754 | -0.020 | -0.3207 | No |
| 88 | PRKACA | na | 20814 | -0.020 | -0.3214 | No |
| 89 | RAC2 | na | 20865 | -0.021 | -0.3218 | No |
| 90 | STAT2 | na | 20888 | -0.021 | -0.3214 | No |
| 91 | JAK2 | na | 21143 | -0.023 | -0.3276 | No |
| 92 | CCL1 | na | 21163 | -0.023 | -0.3270 | No |
| 93 | ROCK2 | na | 21182 | -0.024 | -0.3264 | No |
| 94 | CCL21 | na | 21387 | -0.026 | -0.3310 | No |
| 95 | NFKBIB | na | 21812 | -0.030 | -0.3418 | No |
| 96 | CXCL14 | na | 22280 | -0.035 | -0.3536 | No |
| 97 | GRB2 | na | 22294 | -0.035 | -0.3523 | No |
| 98 | IKBKB | na | 22300 | -0.035 | -0.3507 | No |
| 99 | GSK3B | na | 22452 | -0.037 | -0.3533 | No |
| 100 | CXCL16 | na | 22604 | -0.039 | -0.3558 | No |
| 101 | CDC42 | na | 22626 | -0.039 | -0.3545 | No |
| 102 | MAPK1 | na | 22705 | -0.040 | -0.3548 | No |
| 103 | IKBKG | na | 23063 | -0.044 | -0.3630 | No |
| 104 | ADCY8 | na | 23069 | -0.044 | -0.3609 | No |
| 105 | CRK | na | 23147 | -0.045 | -0.3610 | No |
| 106 | ADCY3 | na | 23170 | -0.045 | -0.3594 | No |
| 107 | GNB3 | na | 23215 | -0.046 | -0.3584 | No |
| 108 | XCL1 | na | 23361 | -0.048 | -0.3603 | No |
| 109 | GRK5 | na | 23415 | -0.048 | -0.3594 | No |
| 110 | FOXO3 | na | 23455 | -0.049 | -0.3582 | No |
| 111 | PTK2B | na | 23645 | -0.052 | -0.3611 | No |
| 112 | CCL16 | na | 23920 | -0.055 | -0.3663 | No |
| 113 | SHC1 | na | 23972 | -0.056 | -0.3651 | No |
| 114 | CCR1 | na | 24035 | -0.057 | -0.3641 | No |
| 115 | CXCL2 | na | 24095 | -0.058 | -0.3630 | No |
| 116 | PTK2 | na | 24353 | -0.061 | -0.3674 | No |
| 117 | CXCR6 | na | 24515 | -0.063 | -0.3690 | No |
| 118 | GNG12 | na | 24895 | -0.069 | -0.3766 | No |
| 119 | PIK3CD | na | 24971 | -0.070 | -0.3753 | No |
| 120 | CCR8 | na | 25084 | -0.072 | -0.3750 | No |
| 121 | CCL8 | na | 25104 | -0.072 | -0.3720 | No |
| 122 | GNG11 | na | 25142 | -0.073 | -0.3695 | No |
| 123 | STAT3 | na | 25462 | -0.078 | -0.3749 | No |
| 124 | GNAI1 | na | 25682 | -0.082 | -0.3773 | No |
| 125 | NFKB1 | na | 25842 | -0.084 | -0.3777 | No |
| 126 | CCR3 | na | 26257 | -0.093 | -0.3852 | No |
| 127 | CXCL12 | na | 26671 | -0.101 | -0.3922 | Yes |
| 128 | PRKACB | na | 26730 | -0.102 | -0.3889 | Yes |
| 129 | CXCL8 | na | 26911 | -0.106 | -0.3889 | Yes |
| 130 | CRKL | na | 27010 | -0.108 | -0.3864 | Yes |
| 131 | NRAS | na | 27014 | -0.108 | -0.3812 | Yes |
| 132 | GNG2 | na | 27033 | -0.109 | -0.3764 | Yes |
| 133 | NCF1 | na | 27044 | -0.109 | -0.3714 | Yes |
| 134 | CCL28 | na | 27313 | -0.115 | -0.3735 | Yes |
| 135 | PIK3CG | na | 27455 | -0.118 | -0.3718 | Yes |
| 136 | NFKBIA | na | 27480 | -0.118 | -0.3667 | Yes |
| 137 | PAK1 | na | 28053 | -0.132 | -0.3767 | Yes |
| 138 | CXCL13 | na | 28109 | -0.134 | -0.3718 | Yes |
| 139 | PRKACG | na | 28125 | -0.134 | -0.3656 | Yes |
| 140 | LYN | na | 28223 | -0.136 | -0.3618 | Yes |
| 141 | PREX1 | na | 28260 | -0.137 | -0.3561 | Yes |
| 142 | CXCR3 | na | 28309 | -0.138 | -0.3507 | Yes |
| 143 | GNG4 | na | 28333 | -0.139 | -0.3446 | Yes |
| 144 | RELA | na | 28616 | -0.147 | -0.3456 | Yes |
| 145 | VAV1 | na | 28859 | -0.153 | -0.3451 | Yes |
| 146 | CCL13 | na | 28966 | -0.156 | -0.3405 | Yes |
| 147 | ITK | na | 29143 | -0.161 | -0.3377 | Yes |
| 148 | PXN | na | 29171 | -0.162 | -0.3306 | Yes |
| 149 | CCR9 | na | 29179 | -0.162 | -0.3228 | Yes |
| 150 | MAP2K1 | na | 29246 | -0.164 | -0.3167 | Yes |
| 151 | CXCL11 | na | 29277 | -0.165 | -0.3094 | Yes |
| 152 | GNGT1 | na | 29309 | -0.166 | -0.3022 | Yes |
| 153 | CCL19 | na | 29544 | -0.174 | -0.3004 | Yes |
| 154 | CCL14 | na | 29709 | -0.179 | -0.2964 | Yes |
| 155 | CCR7 | na | 29717 | -0.180 | -0.2878 | Yes |
| 156 | ADCY1 | na | 29881 | -0.185 | -0.2834 | Yes |
| 157 | GRK4 | na | 30031 | -0.189 | -0.2785 | Yes |
| 158 | CXCL3 | na | 30419 | -0.201 | -0.2798 | Yes |
| 159 | CX3CR1 | na | 30440 | -0.202 | -0.2705 | Yes |
| 160 | CXCL6 | na | 30806 | -0.214 | -0.2705 | Yes |
| 161 | GNGT2 | na | 30927 | -0.218 | -0.2633 | Yes |
| 162 | CCL20 | na | 31140 | -0.226 | -0.2584 | Yes |
| 163 | GNB4 | na | 31197 | -0.228 | -0.2488 | Yes |
| 164 | CCL3 | na | 31224 | -0.229 | -0.2383 | Yes |
| 165 | CCL7 | na | 31858 | -0.256 | -0.2441 | Yes |
| 166 | PLCB1 | na | 32023 | -0.265 | -0.2358 | Yes |
| 167 | CCL25 | na | 32045 | -0.266 | -0.2234 | Yes |
| 168 | CCL5 | na | 32225 | -0.277 | -0.2150 | Yes |
| 169 | GRK1 | na | 32469 | -0.291 | -0.2077 | Yes |
| 170 | SHC2 | na | 32639 | -0.303 | -0.1977 | Yes |
| 171 | CX3CL1 | na | 32800 | -0.314 | -0.1870 | Yes |
| 172 | CCL22 | na | 32998 | -0.328 | -0.1766 | Yes |
| 173 | CXCR4 | na | 33022 | -0.330 | -0.1611 | Yes |
| 174 | SHC4 | na | 33253 | -0.349 | -0.1506 | Yes |
| 175 | CXCL1 | na | 33274 | -0.350 | -0.1340 | Yes |
| 176 | CXCR1 | na | 33350 | -0.357 | -0.1187 | Yes |
| 177 | AKT3 | na | 33656 | -0.385 | -0.1086 | Yes |
| 178 | CCL27 | na | 33678 | -0.388 | -0.0902 | Yes |
| 179 | GRK7 | na | 33698 | -0.390 | -0.0716 | Yes |
| 180 | PRKCB | na | 34153 | -0.452 | -0.0626 | Yes |
| 181 | DOCK2 | na | 34391 | -0.511 | -0.0444 | Yes |
| 182 | CXCL9 | na | 34407 | -0.517 | -0.0195 | Yes |
| 183 | CXCL10 | na | 34557 | -0.586 | 0.0049 | Yes |
Table: GSEA details [plain text format]

  

Fig 2: KEGG\_CHEMOKINE\_SIGNALING\_PATHWAY      
 Blue-Pink O' Gram in the Space of the Analyzed GeneSet

  

Fig 3: KEGG\_CHEMOKINE\_SIGNALING\_PATHWAY: Random ES distribution      
 Gene set null distribution of ES for **KEGG\_CHEMOKINE\_SIGNALING\_PATHWAY**
